# Acute intense fatigue does not modify the effect of EVA and TPU custom foot orthoses on running mechanics, running economy and perceived comfort

**DOI:** 10.1007/s00421-022-04903-9

**Published:** 2022-02-24

**Authors:** Ken Van Alsenoy, Joong Hyun Ryu, Olivier Girard

**Affiliations:** 1 Aspetar, Orthopaedic and Sports Medicine Hospital, FIFA Medical Centre of Excellence, Doha, Qatar; 2 Centre for Health, Activity and Rehabilitation Research (CHEARR), Queen Margaret University, Edinburgh, UK; 3 Sports Science Department, Aspire Academy, Doha, Qatar; 4 School of Human Sciences (Exercise and Sport Science), The University of Western Australia, Perth, WA Australia

**Keywords:** Fatigue, Orthotics, Material resilience, Stride pattern, Economy of locomotion, Footwear comfort

## Abstract

We determined whether fatigue modifies the effect of custom foot orthoses manufactured from ethyl-vinyl acetate (EVA) and expanded thermoplastic polyurethane (TPU) materials, both compared to standardized footwear (CON), on running mechanics, running economy, and perceived comfort. Eighteen well-trained, males ran on an instrumented treadmill for 6 min at the speed corresponding to their first ventilatory threshold (13.8 ± 1.1 km/h) in three footwear conditions (CON, EVA, and TPU). Immediately after completion of a repeated-sprints exercise (8 × 5 s treadmill sprints, rest = 25 s), these run tests were replicated. Running mechanics, running economy and perceived comfort were determined. Two-way repeated measures ANOVA [condition (CON, EVA, and TPU) × fatigue (fresh and fatigued)] were conducted. Flight time shortened (*P* = 0.026), peak braking (*P* = 0.016) and push-off (*P* = 0.032) forces decreased and vertical stiffness increased (*P* = 0.014) from before to after the repeated-sprint exercise, independent of footwear condition. There was a global fatigue-induced deterioration in running economy (− 1.6 ± 0.4%; *P* < 0.001). There was no significant condition × fatigue [except mean loading rate (*P* = 0.046)] for the large majority of biomechanical, cardio-respiratory [except minute ventilation (*P* = 0.020) and breathing frequency (*P* = 0.019)] and perceived comfort variables. Acute intense fatigue does not modify the effect of custom foot orthoses with different resilience characteristics on running mechanics, running economy and perceived comfort.

## Introduction

Custom foot orthoses (CFOs), which refer to shoe inserts built from a three-dimensional representation of the athlete’s feet, have become a contemporary topic in footwear biomechanics literature. Wearing CFOs is used to provide foot support and shock absorption during ground contact through re-distribution of plantar loading and a better maintenance of foot stability (Crago et al. 2019). Prior studies investigating CFOs effects on key biomechanical indicators have reported mixed results with beneficial (Worobets et al. 2014; Wilkinson et al. 2018) or unchanged (Lewinson et al. 2013) adjustments in stride pattern. Discrepant findings may relate to intrinsic properties (i.e., energy return and longitudinal bending stiffness) of tested insoles provoking specific biomechanical modifications in running gait (Sinclair et al. 2016). Quantifying stride mechanical adjustments in response to CFOs is crucial to better understand how footwear features may eventually influence the energetic cost of running (Moore 2016).

Running economy (RE), the steady-state oxygen uptake at a constant submaximal speed, is considered a key physiological measure for distance runners (Barnes and Kilding 2015). The effect of inserts (foot orthoses and sock absorbing insoles) on RE in distance runners has produced inconsistent results (Crago et al. 2019). In a study by Burke and Papuga (2012), six recreational athletes consumed at least 3% less oxygen while running with CFOs compared to their shoe-fitted insoles. In contrast, the effect of wearing CFOs manufactured from ethyl-vinyl acetate (EVA) and expanded thermoplastic polyurethane (TPU) materials, both compared to standardized (shoe only), on RE was considered negligible and marginally improved (albeit not significantly), respectively (Van Alsenoy et al. 2019). Additionally, it is known that the amount of cushioning material in the shoe can influence RE (Tung et al. 2014), and that these effects might be mediated by comfort perception (Mundermann et al. 2003).

Footwear comfort is paramount since it largely influences the adherence to ongoing use of CFOs. The perception of load attenuation (i.e., perceived comfort), resulting from the level of somatosensory feedback experienced (Robbins and Hanna 1987), has been related to RE (Luo et al. 2009; Lindorfer et al. 2020). Comfort-induced changes, as a result of load attenuation on certain anatomical foot structures, potentially contribute to reduction in metabolic demands via more economical stride characteristics (Moore et al. 2014). Analyzing the runner’s perception of the CFOs’ cushioning properties and the associated biomechanical adjustments induced by different inserts features (yet with identical geometry) may also help to better elucidate their effectiveness at protecting against fatigue effects (Hintzy et al. 2015).

Tolerance to ground impact is often compromised as fatigue appears, which in turn may limit performance and/or increase injury risk, notably by increasing the magnitude and rate of loading (Li et al. 2020). To date, little attention has been paid to the effectiveness of CFOs at reducing impact loading in situations of intense fatigue (i.e., repeated ‘all out’ efforts; Girard et al. 2020), with participants typically tested in ‘fresh’ conditions only. Reportedly, CFOs reduced plantar loading under the hallux, medial midfoot, and lateral midfoot compared to prefabricated insoles by ~ 30–35% post-fatigue (12 min at treadmill speed of ~ 14.4 km/h) (Lucas-Ceuvas et al. 2014). It is therefore plausible that the use of CFOs may become a more important protective mechanism for excessive mechanical constraints in the lower extremities once runners become fatigued. Because increased fatigue differently affects the biomechanical pattern of running (Brocherie et al. 2016), assessing the effects of CFOs for spatio-temporal, spring-mass model and antero-posterior variables is relevant.

This study determined whether acute intense fatigue modifies the effect of CFOs manufactured from ethyl-vinyl acetate (EVA) and expanded thermoplastic polyurethane (TPU) materials, both compared to standardized footwear (CON), on running mechanics, RE, and perceived comfort.

## Methods

### Participants

Eighteen male well-trained athletes (mean ± SD age, 38.9 ± 5.1 years; body height, 175.3 ± 5.8 cm; body mass 74.9 ± 7.7 kg; maximal oxygen uptake, 49.1 ± 6.6 mL/min/kg; maximal aerobic speed, 18.4 ± 1.6 km/h) were recruited for this study. They trained on average 8.8 ± 3.7 h per week in the 3 months leading up to the data collection with an average weekly running distance of 37.6 ± 26.7 km. Thirteen were rear-foot strikers, one was a midfoot striker and four were forefoot strikers at 10 km/h. Written informed consent was obtained from participants, and the study was approved by Anti-Doping Laboratory Ethics Committee in Qatar (IRB Application Number 2017000201) and conducted according to the Declaration of Helsinki.

### Study design

About 1 week before testing, participants undertook a preliminary session. They completed a continuous, maximal incremental running test where the individual ventilatory threshold, and corresponding running speed that was used for the three following intervention sessions, were determined. Briefly, participants started running at 9 km/h with speed increases of 0.5 km/h every 30 s. The test ended with voluntary exhaustion of the participants. Verbal encouragement was only given by the researcher guiding the runners throughout the session. Ventilatory threshold was determined using the criteria of an increase in minute ventilation/oxygen uptake with no increase in minute ventilation/carbon dioxide and the departure from linearity of minute ventilation (Davis 1985).

On three occasions, participants performed (in a counter-balanced randomized crossover design), at the same time of day (± 1 h) and 4–5 days apart, an exercise protocol (see below) in different footwear conditions: a control session where participants ran with standardized (i.e., only shoe liner inserted) footwear, CFOs made of EVA and TPU. After arrival to the laboratory, CFOs were inserted bilaterally in participants’ shoes. The participants and the researcher who was directly involved in guiding the session were visually blinded from the CFO materials. Participants were asked to avoid strenuous exercise in the 12 h, as well as refrain from food and caffeine for 4 h preceding their visits to the laboratory and were encouraged to replicate their diet and training pattern for all visits. Laboratory conditions were similar throughout all running sessions (mean ± SD temperature 20.7 ± 0.2 °C, relative humidity 60.4 ± 0.6%). Time of day was standardized for each participant over all sessions.

### Exercise protocol

After a 10 min warm-up at 10 km/h, followed by a 3 min break used to put on the mask to collect expired gases, participants ran for 6 min at the speed associated with their first ventilatory threshold (13.8 ± 1.1 km/h) whereby running mechanics, RE and perceived comfort were evaluated. Participants were then allowed 5 min to rest in a standing position prior to undertaking a fatiguing task that consisted of performing eight, 5 s sprints separated by 25 s of rest (Girard et al. 2020). Lastly, 2 min after the termination of the fatiguing task, participants repeated the 6 min run trial. The complete timing sequence from warm-up to finish was strictly controlled and guided by visual and verbal cues.

### Footwear

During all running, the participants used neutral-like running shoes (Pearl Izumi N2v2, Colorado, US) with an average European shoe size of 43.6 ± 1.6, a stack height of 23–24 mm and a heel drop of 4 mm. The two pairs of CFOs used by participants were based on an individual non-weight-bearing 3D scan of the foot using a Delcam iCube scanner (Elinvision, Karmėlava, Lithuania). CFOs were designed by a sport podiatrist with nearly 20 years of experience, using the Orthomodel Pro CAD software (Autodesk, California, USA). Briefly, scans were imported into the software, markers were placed over the heel, first- and fifth metatarsal and medial arch. A base model surface was adjusted to match the contour of the foot using cross-sectional views from the heel to the forefoot. The thickness of the orthotic was arbitrary set to 8 mm in an attempt to maximize the potential of the TPU beats inside the Infinergy^®^ material (BASF, Ludwigshafen, Germany). All CFOs were direct-milled out of EVA and TPU materials and manually finished to fit inside the shoes. Wear-in time between the first and second intervention session was 4.5 ± 2.5 days and 4.6 ± 2.8 days between the second and last intervention session. The mass of the three footwear conditions was on average 600.3 ± 32.0 g, 647.3 ± 36.0 g and 681.1 ± 35.7 g for the shoes with its original liners (CON), with the custom EVA orthoses (EVA) and with the custom TPU orthoses (TPU), respectively.

### Running mechanics

An instrumented treadmill [ADAL3D-WR, Medical Development—HEF Tecmachine, France; for details, see Belli et al. (2001)] was used for all running conditions. Briefly, it is mounted on a highly rigid metal frame, set at 0°grade incline, fixed to the ground through four piezoelectric force transducers (KI 9077b; Kistler, Winterthur, Switzerland) and installed on a specially engineered concrete slab to ensure maximal rigidity of the supporting ground (Girard et al 2017). In this study, the treadmill function was switched to either constant speed mode (i.e., to measure the constant speed running pattern with direct ground reaction force measurement) or constant motor torque mode (i.e., to allow participants to perform sprints; Morin et al. 2010).

Over the last 2 minutes of each 6 min run, three-dimensional ground reaction force was continuously sampled at 1000 Hz. Ten consecutive steps recorded after running for ~ 4 min 15 s, ~ 4 min 45 s, ~ 5 min 15 s and ~ 5 min 45 s were subsequently averaged for final analysis. After appropriate filtering (Butterworth-type 30 Hz low-pass filter), instantaneous data of vertical and antero-posterior ground reaction forces were averaged for each support phase when the vertical force was above 30 N. These data were determined by measurement of the main spatio-temporal variables: contact time (s), flight time (s) and step frequency (Hz) were reported. Peak braking and peak push-off forces (BW) along with duration of braking and push-off phases (s) were determined. Finally, average vertical loading rate (BW/s) was calculated as the mean value of the time-derivate of vertical force signal within the first 50 ms of the support phase (Li et al. 2020).

A linear spring-mass model paradigm was used to investigate the main mechanical integrative variables characterizing the lower limb behavior during running (McMahon and Cheng 1990). Vertical stiffness (kN/m) was calculated as the ratio of peak vertical forces (N) to the maximal vertical downward displacement of center of mass (m), which was determined by double integration of vertical acceleration of center of mass over time during ground contact (Cavagna 1975). Leg stiffness (kN/m) was calculated as the ratio of peak vertical forces to the maximum leg spring compression [maximal vertical downward displacement + L_0_-√L_0_^2^–(0.5 × running speed × contact time)^2^, in m], both occurring at mid-stance (Morin et al. 2005). Initial leg length (L_0_, great trochanter to ground distance in a standing position) was determined from participant’s stature as L_0_ = 0.53 × stature (Morin et al. 2005).

### Cardio-respiratory variables

Expired gases were collected by a metabolic cart (Jeager™ Oxycon Mobile, Carefusion, Hoechberg, Germany). Prior to each session, calibration of gas sensor was completed for ambient air and a known gas mixture (16% oxygen, 5% carbon dioxide). Turbine was calibrated using a 3 Liter (± 0.4%) syringe and automated high and low flow ventilation. Breath-by-breath gas samples were first averaged every 15 s and subsequently expressed as the average of the last 2 minutes of each 6 min run. Oxygen uptake expressed in both absolute (mL/min) and relative (mL/kg/min) terms, minute ventilation (L/min), breathing frequency (breaths/min), tidal volume (L) were determined. Heart rate (beats/min) was continuously measured by short-range telemetry (Polar, Kempele, Finland). RE was calculated as the oxygen uptake per body mass over speed, expressed in milliliters of oxygen consumed per kilogram per kilometer (mL/kg/km). The metabolic cart was suspended from the ceiling next to participants, so they did not have to support the additional weight of the system when running.

### Perceptual and comfort measures

Within the first minute after finishing low-speed and high-speed runs, a global (6 min run) rating of perceived exertion value was collected using the 6–20 Borg scale. A modified version of the footwear comfort assessment tool, developed and tested on reliability by Mundermann (2002), was used to assess comfort associated with wearing each footwear condition using an iPad mini (Apple, California, US). This scale was used in previous studies to assess perceived comfort (McPoil et al. 2011; Burke and Papuga 2012). For this study, only six of the nine items (‘overall comfort’, ‘heel cushioning’, ‘forefoot cushioning’, ‘medio-lateral control’, ‘arch height’ and ‘heel cup fit’) were scored on a digital, 150 mm visual analogic scale where 0 was defined as ‘not comfortable at all’ and 150 ‘most comfortable condition imaginable’.

### Statistical analysis

Values are presented as mean ± SD. Two-way repeated measures analysis of variance (ANOVAs) [Condition (CON, EVA, TPU) × Fatigue (fresh and fatigued)] were used to compare investigated variables. To assess assumptions of variance, Mauchly’s test of sphericity was performed using all ANOVA results. A Greenhouse–Geisser correction was performed to adjust the degree of freedom if an assumption was violated, while post hoc pairwise-comparisons with Bonferroni-adjusted *P* values were performed if a significant main effect was observed. Partial eta-squared (*η*_p_^2^, with *η*_p_^2^ ≥ 0.06 representing a moderate effect and *η*_p_^2^ ≥ 0.14 a large effect) values were calculated. All statistical calculations were performed using SPSS statistical software V.26.0 (IBM Corp., Armonk, USA). The significance level was set at *P* < 0.05.

## Results

### Running mechanics (Table 1)

There was a significant main condition effect for mean loading rate, push-off duration and push-off peak force (all *P* ≤ 0.017; 0.27 ≤ *η*_p_^2^ ≤ 0.47). A significant main fatigue effect was noted for four out of nine variables studied: flight time, vertical stiffness, as well as braking and push-off durations (all *P* ≤ 0.032; 0.26 ≤ *η*_p_^2^ ≤ 0.32). There was no significant condition × fatigue [except mean loading rate (*P* = 0.046; *η*_p_^2^ = 0.19)] interactions for any stride mechanical variable.

**Table 1:** Changes in biomechanical parameters for shoe only (CON), shoe with Ethyl-Vinyl Acetate orthotic (EVA), and shoe with Thermoplastic Poly-Urethane orthotic (TPU) conditions before (Fresh) and after (Fatigued) the completion of a repeated-sprint treadmill exercise

Variables	CON	EVA	TPU	ANOVA *P* value (*η*^2^)		
				C	F	C × F
Contact time (ms)						
Fresh	223 ± 17	225 ± 17	226 ± 17	0.121	0.332	0.404
Fatigued	225 ± 18	225 ± 18	226 ± 17	(0.12)	(0.06)	(0.06)
Flight time (ms)						
Fresh	117 ± 18	116 ± 19	117 ± 17	0.354	**0.026**	0.571
Fatigued	112 ± 18*	113 ± 17*	115 ± 17*	(0.06)	(0.27)	(0.03)
Step frequency (Hz)						
Fresh	2.97 ± 0.15	2.96 ± 0.14	2.94 ± 0.14	0.076	0.069	0.953
Fatigued	2.95 ± 0.15	2.94 ± 0.12	2.94 ± 0.12	(0.15)	(0.19)	(0.01)
Mean loading rate (BW/s)						
Fresh	62.3 ± 13.8†	62.4 ± 14.0†	55.0 ± 10.6	** < 0.001**	0.684	**0.046**
Fatigued	60.4 ± 14.1†*	62.2 ± 14.6†	56.1 ± 12.3	(0.46)	(0.01)	(0.19)
Vertical stiffness (kN/m)						
Fresh	34.8 ± 4.5	35.5 ± 4.3	35.2 ± 4.6	0.617	**0.014**	0.878
Fatigued	36.0 ± 5.3*	36.4 ± 4.5*	36.4 ± 5.0*	(0.02)	(0.32)	(0.01)
Leg stiffness (kN/m)						
Fresh	15.7 ± 2.5	15.6 ± 2.2	15.5 ± 2.2	0.88	0.788	0.152
Fatigued	15.4 ± 2.1	15.5 ± 2.1	15.7 ± 1.9	(0.01)	(0.01)	(0.11)
Braking phase duration (ms)						
Fresh	109 ± 9	109 ± 8	110 ± 8	0.828	0.423	0.32
Fatigued	110 ± 9	110 ± 8	109 ± 7	(0.01)	(0.040)	(0.07)
Push-off phase duration (ms)						
Fresh	114 ± 11†	115 ± 12	116 ± 12	**0.003**	0.309	0.368
Fatigued	115 ± 12†	115 ± 13	117 ± 12	(0.31)	(0.06)	(0.06)
Peak braking force (kN)						
Fresh	0.59 ± 0.11	0.58 ± 0.13	0.60 ± 0.11	0.253	**0.016**	0.895
Fatigued	0.58 ± 0.11*	0.57 ± 0.13*	0.59 ± 0.11*	(0.08)	(0.31)	(0.01)
Peak push-off force (kN)						
Fresh	0.41 ± 0.06†	0.39 ± 0.07	0.39 ± 0.06	**0.017**	**0.032**	0.867
Fatigued	0.40 ± 0.06†*	0.39 ± 0.06*	0.38 ± 0.06*	(0.27)	(0.26)	(0.1)

Bold values indicate statistically significant ANOVA *P* values (*P* < 0.05)

Values are mean ± SD. C and F, respectively, refer to ANOVA main effects of condition and fatigue and interaction between these two factors with *P* value and partial eta-squared (*η*^2^) in parentheses. Bold values indicate statistically significant findings

^†^Significantly different from TPU, *P* < 0.05

*Significantly different from Fresh, *P* < 0.05

### Cardio-respiratory variables (Table 2)

There was a significant main condition effect for heart rate (*P* = 0.027; *η*_p_^2^ = 0.19) only. All examined cardiorespiratory variables changed significantly from fresh to fatigued state (all *P* ≤ 0.021; 0.28 ≤ *η*_p_^2^ ≤ 0.83), except tidal volume (*P* = 0.507; *η*_p_^2^ = 0.03). Only minute ventilation (*P* = 0.020; *η*_p_^2^ = 0.21) and breathing frequency (*P* = 0.019; *η*_p_^2^ = 0.21) displayed significant condition × fatigue interactions.

**Table 2:** Changes in cardio-respiratory parameters for shoe only (CON), shoe with Ethyl-Vinyl Acetate orthotic (EVA), and shoe with Thermoplastic Poly-Urethane orthotic (TPU) conditions before (Fresh) and after (Fatigued) the completion of a repeated-sprint treadmill exercise

Variables	CON	EVA	TPU	ANOVA *P* value *(η*^*2*^*)*		
				C	F	C × F
Heart rate (bpm)						
Fresh	164 ± 13†	163 ± 13	162 ± 13	**0.027**	** < 0.001**	0.904
Fatigued	172 ± 13†*	171 ± 12*	170 ± 13*	(0.19)	(0.60)	(0.01)
Running economy (mL/kg/km)						
Fresh	190 ± 11	185 ± 14	188 ± 11	0.077	**0.020**	0.746
Fatigued	193 ± 12*	189 ± 14*	191 ± 13*	(0.14)	(0.28)	(0.02)
Oxygen uptake (mL/kg/min)						
Fresh	43.7 ± 4.0	42.5 ± 4.5	43.4 ± 5.0	0.087	**0.021**	0.796
Fatigued	44.4 ± 4.8*	43.4 ± 4.5*	44.0 ± 5.5*	(0.13)	(0.28)	(0.02)
Minute ventilation (L/min)						
Fresh	101 ± 16	101 ± 16	100 ± 15	0.058	** < 0.001**	**0.020**
Fatigued	119 ± 20†*	116 ± 21†*	112 ± 19*	(0.15)	(0.71)	(0.21)
Breathing frequency (breath/min)						
Fresh	42.6 ± 6.8	44.9 ± 10.7	44.5 ± 8.6	*0.496*	** < 0.001**	**0.019**
Fatigued	50.9 ± 8.4†*	50.2 ± 8.9*	49.0 ± 8.3*	(0.03)	(0.83)	(0.21)
Tidal volume (L)						
Fresh	2.39 ± 0.43	2.31 ± 0.57	2.30 ± 0.48	(*0.204*)	0.507	0.420
Fatigued	2.37 ± 0.50	2.35 ± 0.56	2.33 ± 0.51	(0.09)	(0.03)	(0.05)

Bold values indicate statistically significant ANOVA *P* values (*P* < 0.05)

Values are mean ± SD. C and F, respectively, refer to ANOVA main effects of condition and fatigue and interaction between these two factors with *P* value and partial eta-squared (*η*^2^) in parentheses. Bold values indicate statistically significant findings

^†^Significantly different from TPU, *P* < 0.05

*Significantly different from Fresh, *P* < 0.05

### Perceptual and Comfort measures (Table 3)

There was a significant main condition effect for medio-lateral control and arch height (all *P* ≤ 0.027; 0.20 ≤ *η*_p_^2^ ≤ 0.30). Increased ratings of perceived exertion (*P* < 0.001; *η*_p_^2^ = 0.71) occurred under fatigue, while heel cushioning (*P* = 0.021; *η*_p_^2^ = 0.29) was also rated as more comfortable.

**Table 3:** Changes in rating of perceived exertion (RPE) and comfort parameters for shoe only (CON), shoe with Ethyl-Vinyl Acetate orthotic (EVA), and shoe with Thermoplastic Poly-Urethane orthotic (TPU) conditions before (Fresh) and after (Fatigued) the completion of a repeated-sprint treadmill exercise

Variables	CON	EVA	TPU	ANOVA *P* value *(η*^*2*^*)*		
				C	F	C × F
RPE						
Fresh	12.7 ± 3.1	13.2 ± 3.1	13.0 ± 3.0	0.256	** < 0.001**	0.738
Fatigued	14.7 ± 4.2*	15.1 ± 3.1*	13.7 ± 3.1*	(0.08)	(0.71)	(0.01)
Overall comfort						
Fresh	86 ± 32	93 ± 31	97 ± 25	0.089	0.163	0.121
Fatigued	81 ± 31	105 ± 21	101 ± 23	(0.15)	(0.12)	(0.14)
Heel cushioning						
Fresh	83 ± 33	96 ± 23	89 ± 25	0.090	**0.021**	0.813
Fatigued	85 ± 27*	102 ± 22*	93 ± 26*	(0.15)	(0.29)	(0.01)
Forefoot cushioning						
Fresh	88 ± 34	96 ± 28	102 ± 23	0.079	0.326	0.086
Fatigued	84 ± 29	105 ± 24	104 ± 22	(0.16)	(0.06)	(0.16)
Medio-lateral control						
Fresh	83 ± 32^#^†	98 ± 26	101 ± 25	**0.027**	0.080	0.361
Fatigued	84 ± 30^#^†	107 ± 22	100 ± 24	(0.20)	(0.18)	(0.06)
Arch height						
Fresh	74 ± 36^#^†	92 ± 33	96 ± 24	**0.009**	0.346	0.060
Fatigued	72 ± 32^#^†	102 ± 28	95 ± 26	(0.30)	(0.06)	(0.18)
Heel cup fit						
Fresh	86 ± 29	95 ± 23	88 ± 29	0.135	0.206	0.416
Fatigued	85 ± 29	102 ± 22	93 ± 26	(0.12)	(0.10)	(0.05)

Bold values indicate statistically significant ANOVA *P* values (*P* < 0.05)

RPE was assessed using a 6–20 Borg scale and other comfort parameters were measures using a Visual Analog Scale (0–150 mm); Values are mean ± SD. Values are mean ± SD. C and F, respectively, refer to ANOVA main effects of condition and fatigue and interaction between these two factors with *P* value and partial eta-squared (*η*^2^) in parentheses. Bold values indicate statistically significant findings

^#^Significantly different from EVA, *P* < 0.05

^†^Significantly different from TPU, *P* < 0.05

*Significantly different from Fresh, *P* < 0.05

## Discussion

### Running mechanics

One strength of our study is that running mechanics during constant submaximal runs were derived from direct ground reaction forces’ recording in both vertical and antero-posterior directions, as opposed to previous studies using tri-axial accelerometers to assess the effects of CFOs before and after an intense run (Lucas-Cuevas et al. 2014; Lucas-Cuevas et al. 2017). Lower mean loading rates were recorded while wearing CFO made of TPU compared to EVA or no insert (CON), likely due to the higher resilience material properties (not measured). Contrary to the present study, previous studies observed lower loading rates in stiffer shoes (Henning et al. 1996; Milani et al. 1997). The often-used statement that, based upon their perception, runners adapt their technique with stiffer footwear to avoid high impact rates over the heel by cushioning the ground is not supported.

Biomechanical manifestation of fatigue resulting from completion of a repeated-sprint treadmill exercise was generally not modified by inserts. Out of nine biomechanical variables, only contact time, mean loading rate and braking phase duration displayed statistically significant condition × fatigue interactions, with no systematic advantage of one or the other CFO condition. With fatigue, however, wearing CFOs (EVA or TPU) only generated subtle kinetic (mean loading rate: 1–2 BW/s) and kinematic (contact time and braking phase duration: 1–3 ms) adjustments compared to CON. It must therefore be questioned whether such small fatigue-related differences between footwear conditions are clinically relevant. Despite different resilience characteristics but similar configuration of tested CFOs, our novel findings indicate that participants who wore CFOs, either made of EVA or TPU materials, produced essentially similar fatigue-induced adjustments in their stride pattern at constant treadmill speed.

Inspection of changes in biomechanical variables induced by the repeated-sprint treadmill exercise indicates that globally running technique is not profoundly modified under acute intense fatigue. Regardless of footwear condition, the runners mainly reduced their flight time and increased their vertical stiffness to maintain treadmill speed constant, while the magnitude of these adjustments also did not differ between the two running speeds. Previously, both low (10 km/h) and high (20 km/h) constant speed running patterns were found unchanged from before to ~ 3 min after repeated-sprint exercises [four sets of five, 6 s sprints with 24 s recovery and 3 min between sets (Morin et al. 2012); three sets of five, 5 s sprints with 25 s recovery and 3 min between sets (Girard et al. 2017)] using the ADAL treadmill. Despite exacerbated cardio-respiratory and perceptual (rating of perceived exertion) responses occurring after sprinting repeatedly, tested individuals may have not reached exertion levels where biomechanical manifestation of fatigue would cause more stressful ground impacts.

### Cardio-respiratory variables

As expected, physiological responses were elevated between before and after the fatiguing exercise. No significant interaction was found between fatigue and footwear conditions for key cardio-respiratory variables (i.e., heart rate, oxygen uptake). This finding provides evidence that both types of CFOs (TPU and EVA materials) were not able to protect against fatigue-related deterioration in RE and/or elevations in physiological strain compared to the shoe only (CON) condition. This may not be surprising since no fatigue-induced differences in mechanical variables could be detected across footwear conditions. Similarly, wearing CFOs that alters neuromuscular control during a submaximal 1 h treadmill run was found ineffective to reduce the aerobic cost of running (Kelly et al. 2011). In our study, the biomechanical effect of wearing CFOs with or without fatigue was probably too small to induce meaningful differences in physiological variables (expect minute ventilation that was lower in TPU compared to CON and to a lower extent EVA) across conditions.

Another interesting observation was that RE at a speed corresponding to the first ventilatory threshold deteriorated by ~ 1.7% with fatigue. Results from Day and Hahn (2019) suggest that optimal footwear longitudinal bending stiffness to improve RE in fresh conditions is speed dependent. Whereas most participants running at 14 km/h elicited a minimum metabolic rate in the normal shoe, an increased number of participants were more economical in the stiff shoe (despite it weighing an extra 50 g compared to the normal shoe) at 17 km/h. In our study, EVA and TPU were ~ 50 g (+ 8%) and ~ 80 g (+ 14%) heavier compared to CON, respectively. The relationship between shoe mass and energy cost suggests that energy demands during running are greater as shoe mass is increased (i.e., + 1% for every added 100 g per shoe; Frederick 1984). Consequently, we cannot exclude that the additional weight of inserts may have confounded any protective effect of CFOs on RE when fatigue sets in. While our approach did not account for different footwear mass, imposing running speeds relative to our participants’ physiological capacity rather than absolute speeds, as commonly done in the CFO-related literature on RE (Crago et al. 2019), was a strength.

### Comfort measures

Significantly improved perceived comfort for medio-lateral control (∼20%) and arch height (∼25%) was reported for both EVA and TPU, yet with no difference between the two inserts, compared to CON. Despite statistical significance was not reached, other perceived comfort-related metrics (heel and forefoot cushioning, heel cup fit) displayed similar trends. However, the presumably greater levels of rigidity of the sole of the EVA insert did not generate greater discomfort for the runners, also with similar or improved (e.g., lower loading rates) running biomechanics. Overall, in line with previous research in fresh conditions (Lindorfer et al. 2020), increase in comfort at the foot/shoe interface did not lead to improved RE and meaningful changes in biomechanical variables. Perhaps functional biomechanical variables that were not measured in this study (i.e., plantar pressure distribution, muscle activity, ankle and knee joint moments), known to be influenced by perceived comfort, could explain observed differences between footwear conditions (Dinato et al. 2015). While ground reaction forces are commonly (similar to current approach) used as proxy measurements to reflect biomechanical loads imposed on the lower extremities as a whole, directly quantifying tissue and/or structure-specific strain (i.e., longitudinal arch, Achilles’ tendon) remains a challenge (Verheul et al. 2020).

Unexpectedly, perceived comfort ratings in different regions of the foot were in fact improved after completion of the repeated-sprint treadmill exercise. While the condition × fatigue interaction was not significant, there was a trend for the two CFOs conditions to become more comfortable in the fatigued state compared to CON. Contrastingly, during a 13 km run, decrement in perceived overall footwear comfort became significant only after 44 min of exercise (~ 7.8 km) (Hintzy et al. 2015). Discrepant findings between our results (i.e., assessment before and after a short and intense fatigue protocol) and previous studies (i.e., regular assessments during prolonged running at lower intensity; Hintzy et al. 2015; Jimenez-Perez et al. 2021) could be explained by the methodology used for measuring biomechanical manifestation of fatigue and the nature/degree of fatigue attained by participants. The clinical implication of our findings is that well-trained runners wearing CFOs made of either EVA or TPU materials should not fear deteriorated comfort ratings with acute intense fatigue.

### Limitations

Several limitations must be considered. First, the inclusion of only male runners who were mainly habitual rear-foot strikers (~ 70%). Our findings may not be generalizable to runners with habitual midfoot/forefoot strike patterns and female population since biomechanical variables may differ across various foot strikes and between genders (Moore 2016). Whereas the foot strike pattern of tested athletes was determined, our sample size of 18 participants (with only one and four forefoot and midfoot strikers) was too small to allow meaningful comparisons between groups. Additionally, to reflect ecological situations, participants should undertake over-ground runs with their foot strikes recorded by a number of force plates laid in series. Because inherent characteristics of running shoes per se can alter running mechanics and/or RE (Hoogkamer et al. 2018), participants were not allowed to use their own running shoes. Standardization of footwear conditions across participants (also with the use of personalized inserts) is a strength of our study from a methodological standpoint. Nonetheless, one could speculate that any protective effect of fatigue may have more apparent if individuals were wearing their habitual footwear or other types of foot orthoses.

## Conclusion

Acute intense fatigue does not modify the effect of custom foot orthoses with different resilience characteristics (EVA or TPU materials both compared to standardized footwear) on running mechanics, running economy and perceived comfort. When facing acute intense fatigue, well-trained runners should not expect any protective effects from wearing CFOs.

## Abbreviations

ANOVA: Repeated-measures analysis of variance
CFO: Custom foot orthotics
CON: Control condition
EVA: Ethyl-vinyl acetate
GRF: Ground reaction force
RE: Running economy
RPE: Ratings of perceived exertion
TPU: Thermoplastic polyurethane
